# Tetramine-Based Hyperbranched Polyimide Membranes with Rigid Crosslinker for Improved Gas Permeability and Stability

**DOI:** 10.3390/polym15143017

**Published:** 2023-07-12

**Authors:** Xiangyun Liu, Honglei Ling, Jiangzhou Luo, Xueping Zong, Song Xue

**Affiliations:** 1 School of Materials Science and Engineering, Tianjin University of Technology, Tianjin 300384, China; 2 Tianjin Key Laboratory of Organic Solar Cells and Photochemical Conversion, School of Chemistry and Chemical Engineering, Tianjin University of Technology, Tianjin 300384, China

**Keywords:** hyperbranched polyimide (HBPI), membrane, gas separation, tetramine crosslinking center, DAM comonomer

## Abstract

Triamine-based HBPI membranes are known for high gas separation selectivity and physical stability, but their permeabilities are still very low. In this study, we utilized a tetramine monomer called TPDA (N,N,N′,N′-tetrakis(4-aminophenyl)-1,4-benzenediamine) as a crosslinking center and incorporated an additional diamine comonomer called DAM (2,4,6-trimethyl-1,3-diaminobenzene) to enhance gas separation performance, especially gas permeability. The findings demonstrated that the resultant 6FDA−DAM/TPDA membranes based on tetramine TPDA exhibited a greater amount of free volume compared to the triamine-based HBPI membranes, resulting in significantly higher gas permeabilities. Furthermore, the higher concentration of DAM component led to the generation of more fractional free volumes (FFV). Consequently, the gas permeabilities of the 6FDA−DAM/TPDA membranes increased with an increase in DAM content, with a minimal compromise on selectivity. The enhanced gas permeabilities of the 6FDA−DAM/TPDA membranes enabled them to minimize the footprint required for membrane installations in real-world applications. Moreover, the 6FDA−DAM/TPDA membranes exhibited remarkable durability against physical aging and plasticization, thanks to the incorporation of a hyperbranched network structure.

## 1. Introduction

The energy-efficient technique of membrane-based gas separation emerged as a crucial technique for CO_2_ capture, hydrogen recovery, and natural gas upgrading [1,2,3,4]. The utilization of organic polymeric membrane materials for the fabrication of gas separation membranes has attracted considerable attention in recent research because of its promising economic advantages [5,6,7]. However, three obstacles must be overcome before these membranes can be effectively implemented in industrial plants. These challenges include balancing the trade-off between gas selectivity and permeability, addressing physical aging effects, and mitigating the issue of plasticization [6,8,9].

To restrict physical aging and plasticization in polymeric membranes, researchers have focused on developing three-dimensional network structures. The post-crosslinking methodology has been extensively explored regarding the creation of network-formed polymeric membranes [10,11,12,13]. For instance, in 2013, Koros et al. presented a chemical crosslinking technique applied to linear polyimide membranes. This method involved connecting two polymer chains using PEG crosslinking agents [10]. The researchers successfully suppressed plasticization of the membrane at high feed pressures by this crosslinking approach, and their gas separation performance was also improved. Furthermore, the application of chemical crosslinking has been extended to stabilize hollow fiber membranes, as demonstrated by Liu et al. [12,13]. The resulting crosslinked hollow fiber membranes exhibited exceptional gas separation performance which remained consistently high and stable when exposed to high-pressure and aggressive CO_2_/CH_4_ mixtures.

Besides chemical crosslinking, an alternative and effective method for fabricating network polymeric membranes is through the direct formation of polyimide networks using triamino crosslinking centers [14,15,16,17,18]. In 2001, Okamoto et al. introduced a range of hyperbranched polyimides (HBPI) membranes. These membranes were prepared through the polymerisation of A_2_ (dianhydride) and B_3_ (triamine) components [14]. The HBPI membranes exhibited superior gas pair selectivity compared to their linear counterparts, with CO_2_/N_2_ selectivity of up to 30. The three-dimensional network structure of the HBPI membranes also contributed to their high resistance against physical aging and plasticization. Building upon these advantages, novel HBPI membranes were developed using various A_2_ (dianhydride) and B_3_ (triamine) monomers [17,19,20,21,22]. For example, in 2008, Suzuki et al. prepared a HBPI membrane by polymerizing the dianhydride 6FDA and the triamine TAPOB [20]. This HBPI membrane achieved CO_2_/N_2_ selectivity of 35, although the CO_2_ permeability was only 12.8 Barrer. In 2018, Karel et al. utilized a novel triamine called TTM to produce an ultra-selective HBPI membrane with CO_2_/CH_4_ selectivity of 134 [15]. However, the CO_2_ permeability remained low at 27.4 Barrer. Despite the great stability and remarkably high selectivity of HBPI membranes, their low permeability restricts their competitiveness with other polymeric materials for large-scale gas separations.

To address this permeability issue, various approaches have been investigated to enhance gas permeability of HBPI membranes [18,19,21,23]. For instance, Peter et al. demonstrated that incorporating 4,4′-(hexafluoroisopropylidene) dianiline (6FpDA) into the hyperbranched network can enhance gas permeability. This improvement is attributed to the bulky CF_3_ substituents in the 6FpDA comonomer which increase the distances between polymer chains [18]. In 2020, Deng et al. revealed that adding a contorted comonomer, 1,1′-binaphthyl-2,2′-diamine (BNDA) can increase the polymeric fractional free volumes (FFV), thereby increasing permeability [21]. The highest CO_2_ permeability coefficient reached 73.6 Barrer.

The objective of this study was to further enhance the gas permeability of HBPI membranes while maintaining their anti-aging ability and resistance to plasticization. To achieve this, we made modifications to the crosslinking center and incorporated an additional B_2_-type monomer. Specifically, instead of using B_3_ (triamine) monomers, we opted for a B_4_ (tetramine) monomer called N,N,N′,N′-tetrakis(4-aminophenyl)-1,4-benzenediamine (TPDA) as the crosslinking center. The tertiary amine structure in the TPDA crosslinking center acted as a Lewis base, facilitating increased acid-base interaction between CO_2_ gas molecules and N atoms of the polymer framework. Additionally, the utilization of the tetramine TPDA as a crosslinking center in comparison to the triamine lead to a larger cavity within the network structure. This enlargement contributed to an increased fractional free volume (FFV) of the polymeric material. Furthermore, we incorporated a rigid B_2_ (diamine) comonomer called DAM (2,4,6-trimethyl-1,3-diaminobenzene) into the hyperbranched network to further increase the FFV. The addition of DAM also resulted in the elongation of linear unit in conjunction with the diamine 6FDA monomer, effectively diluting the crosslinking density of the dendritic architecture. This combination of modifications led to a HBPI polymer network with reduced crosslinking density and improved permeability. Importantly, these enhancements in permeability did not compromise the highly selective and stable nature of polymer network, thus resulting in a novel HBPI membrane with improved gas permeability while maintaining desirable properties.

## 2. Experimental

### 2.1. Materials and Chemicals

The substances (4,4-hexafluoroisopropylidene) diphthalic anhydride (6FDA), 2,4,6-trimethyl-1,3-diaminobenzene (DAM), and N,N,N′,N′-tetrakis(4-aminophenyl)-1,4-benzenediamine (TPDA) were purchased from Saeng Chemical Technology (Shanghai) Co., Ltd. (Shanghai, China) Dimethylformamide (DMF) was procured from Tianjin Fu Chen Chemical Reagents Factory (China). Vacuum distillation was employed to purify DMF followed by drying it with molecular sieves. The gases utilized for the permeation measurements were sourced from Beijing Gas Company and had purities exceeding 99.99%.

### 2.2. Synthesis of 6FDA−DAM/TPDA Hyperbranched Polyimides (HBPI) and Membrane Fabrication

**Scheme 1:**
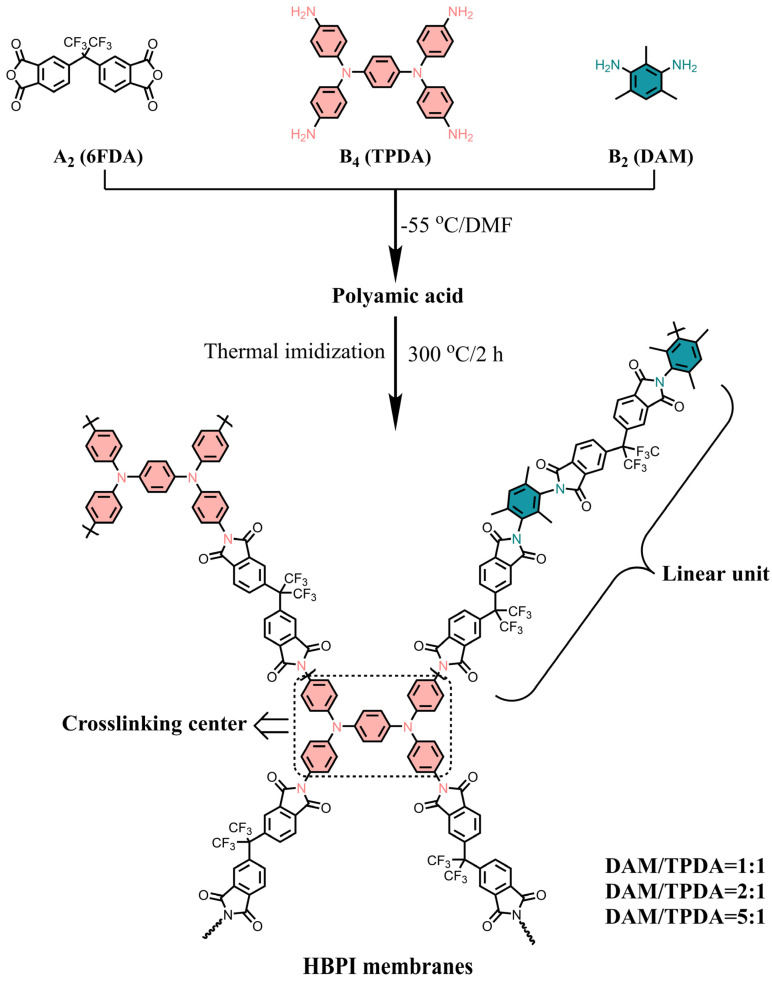
Synthesis route of 6FDA−DAM/TPDA hyperbranched polyimide membranes.

As shown in Scheme 1, 6FDA−DAM/TPDA hyperbranched polyimides (HBPI) were afforded by co-polycondensation between the dianhydride monomer (6FDA), the diamine (DAM), and tetramine (TPDA) monomers. To ensure the formation of high-molecular weight polyimide networks, all monomers (6FDA, DAM and TPDA) were thoroughly dried at 120 °C for 12 h prior to the polymerization process. Different ratios of DAM to TPDA were investigated to achieve optimal gas separation performance in this study.

The synthesis of 6FDA−DAM/TPDA (5:1) followed the typical procedure outlined as follows: TPDA (54.3 mg, 0.11 mmol, 1.0 equivalent) and DAM (83.37 mg, 0.55 mmol, 5.0 equivalents) were added to a dried double-necked flask. The flask was then degassed and purged with nitrogen (N_2_) three times. Under the N_2_ atmosphere, anhydrous DMF (15 mL) was added to the reaction flask and vigorously stirred at room temperature for 15 min until a clear and transparent solution was obtained. Next, the reaction mixture was cooled to −55 °C, and a solution of 6FDA solution (345.2 mg, 0.78 mmol, 7.0 equivalents) in DMF (15 mL) was dropwise added to the reaction. The reaction mixture was vigorously stirred for 3 h, resulting in the formation of a 3.0 wt.% polyamic acid solution.

The polyamic acid solution obtained from the reaction was passed through a PTFE syringe filter and promptly poured onto a levelled petri dish before gelation occurred. The solvent was then gradually evaporated in a vacuum oven at 60 °C over a period of 5 days. Once the solvent evaporated, the resulting 6FDA−DAM/TPDA (5:1) polyamic acid membrane was obtained. To eliminate any remaining solvent, the resulting membrane was then dried at 80 °C for 2 days. The dried polyamic acid membrane was then subjected to thermal imidization by heating it in a 300 °C muffle furnace. This process transformed the polyamic acid into a 6FDA−DAM/TPDA (5:1) hyperbranched polyimide membrane with a thickness of around 20 µm. Using a similar method, other 6FDA−DAM/TPDA membranes with different DAM/TPDA ratios (2:1 and 1:1) were prepared. These membranes were labeled as 6FDA−DAM/TPDA (5:1), 6FDA−DAM/TPDA (2:1), and 6FDA−DAM/TPDA (1:1), respectively.

### 2.3. Characterization

The Fourier transform infrared (FT-IR) spectra of the membranes were acquired using a Perkin Elmer 782 Fourier transform spectrophotometer. The surface and cross-sectional morphologies of the samples were analyzed using a high-resolution field emission scanning electron microscope (SEM, Zeiss, Jena, Germany) operated at 5.0 kV. Wide angle X-ray diffractometry (WAXD) measurements were conducted using a Rigaku Ultima IV X-ray Diffractiometer, and the *d*-spacing values were determined using Bragg’s law. Thermogravimetric analysis (TGA) was conducted using a TA instruments Q500 with a heating rate of 10 °C/min. The mechanical properties of the membranes were assessed using an electronic universal material testing equipment (Changchun Machinery Research Institute Co., Ltd., Changchun, China) with a tensile speed of 0.5 mm/min. X-ray photoelectron spectroscopy (XPS, ESCALAB 250 Xi) with an Al Ka radiator was applied for elemental analysis.

### 2.4. Gas Permeation Measurements

The pure gas permeation measurements were conducted using the constant-volume/variable-pressure method. Circular membranes with an effective exposed surface area of approximately 0.70 cm^2^ were utilized. The gas permeabilities were measured following the order of N_2_, O_2_, and CO_2_ at 30 °C, covering feed pressures ranging from 0.2 to 1.2 MPa. The permeability values were calculated using Equation (1):

(1) $$P=10^{10}\cdot \frac{V_{d}\cdot l}{A{\cdot R\cdot T\cdot P}_{up}}\cdot (\frac{dp}{dt})$$

Here, *P* represents the gas permeability in Barrer (1 Barrer = 10^−10^ cm^3^ (STP) cm cm^−2^ s^−1^ cmHg^−1^), *V_d_* denotes the calibrated permeate volume in cm^3^, l is the membrane thickness (cm), *P_up_* represents the upstream pressure (cmHg), *A* is the effective membrane area (cm^2^), R is the gas constant (0.278 cm^3^cmHg/cm^−3^(STP) K^−1^). T is the operating temperature (K), and *dp/dt* denotes the steady-state permeate-side pressure increase (cmHg/s). The ideal selectivity for a gas pair (*A*/*B*) is calculated as the ratio of their pure-gas permeabilities:

$$\alpha_{A/B}=\frac{P_{A}}{P_{B}}$$

## 3. Results and Discussion

### 3.1. Preparation of 6FDA−DAM/TPDA Membranes

In this study, a two-step method was employed to copolymerize the dianhydride 6FDA with the commercially available diamine DAM and tetramine TPDA. The process involved the formation of a polyamic acid (PAA) solution followed by thermal imidization. It is known that direct polycondensation between tetramine (B_4_) and dianhydride (A_2_) often leads to insoluble gel-like polyimides which hinder the formation of films. To overcome this issue, various approaches have been explored in previous studies, such as slow monomer addition rates [14,15,24] and creating reactivity differences in functional groups [17,25]. In this study, a low-temperature synthesis method previously developed by our group was utilized to control the polymerization reactivities and obtain the initial PAA solution [21]. The PAA solution was then cast into self-standing films and thermally imidized at 300 °C to produce 6FDA−DAM/TPDA membranes. Different ratios of DAM/TPDA comonomers, ranging from 5:1 to 2:1 and 1:1, were investigated in these hyperbranched polyimide (HBPI) membranes. Previous reports have demonstrated that the DAM component can enhance gas permeability by providing a higher fractional free volume (FFV) [12,26].

**Figure 1:**
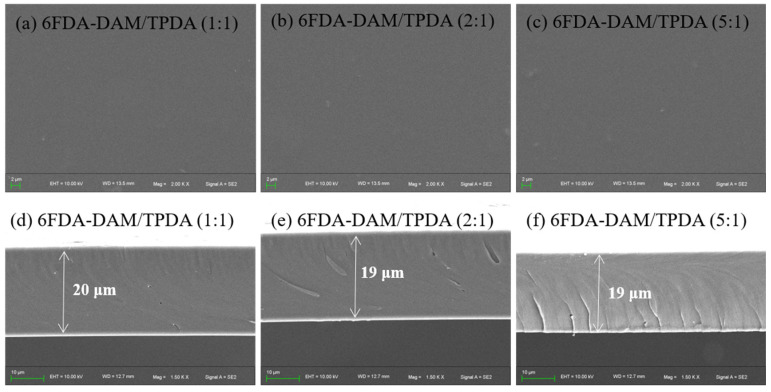
Field-emission scanning electron microscopy (FE-SEM) images of the surface (**a**–**c**) and cross-section (**d**–**f**) of the 6FDA−DAM/TPDA membranes.

As shown in Figure S1, the membranes fabricated through this low-temperature synthesis method exhibited defect-free appearances, with the color gradually changing from dark brown to light brownish with increasing DAM content. Field-emission scanning electron microscopy (FE-SEM) images (Figure 1) confirmed that the 6FDA−DAM/TPDA membranes possessed smooth and dense morphologies, with consistent thickness of 19 or 20 μm.

**Figure 2:**
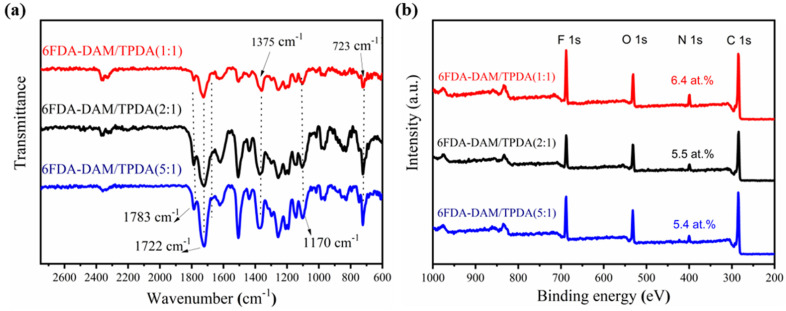
FT-IR (**a**) and XPS (**b**) spectra of 6FDA−DAM/TPDA membranes.

The solubility of the obtained 6FDA−DAM/TPDA membranes was tested by immersing them in organic solvents. As indicated in Table S1, the polyimides were completely insoluble in common solvents such as THF (tetrahydrofuran), DCM (dichloromethane), and DMF (N,N-dimethylformamide), indicating the formation of a network structure. The structural characteristics of the three 6FDA−DAM/TPDA membranes were further analyzed using FT-IR spectroscopy (Figure 2a). The spectra displayed characteristic absorption peaks for 6FDA−DAM/TPDA, including peaks at 1783 cm^−1^ (asymmetric stretching of C=O), 1722 cm^−1^ (symmetric stretching of C=O), 1375 cm^−1^ (stretching vibration of C-N), and 723 cm^−1^ (bending vibration of C=O), confirming the successful formation of six-membered imide rings. Additionally, a peak corresponding to a C-F stretching vibration peak was observed at 1170 cm^−1^, indicating the presence of a CF_3_ group in the 6FDA−DAM/TPDA membranes. Furthermore, XPS was utilized to determine the N content in the 6FDA−DAM/TPDA membranes (Figure 2b). Among the different compositions, the 6FDA−DAM/TPDA (1:1) membrane, which had the highest proportion of tetramine TPDA crosslinking center, exhibited the highest nitrogen content of 6.4%. As the DAM/TPDA ratio increased to 2:1 and 5:1, the nitrogen content decreased to 5.5% and 5.4%, respectively. The decreasing trend of the nitrogen content with increase of DAM/TPDA ratio indicated the successful incorporation of the tetramine TPDA crosslinking center.

### 3.2. Polymer and Membrane Properties

**Figure 3:**
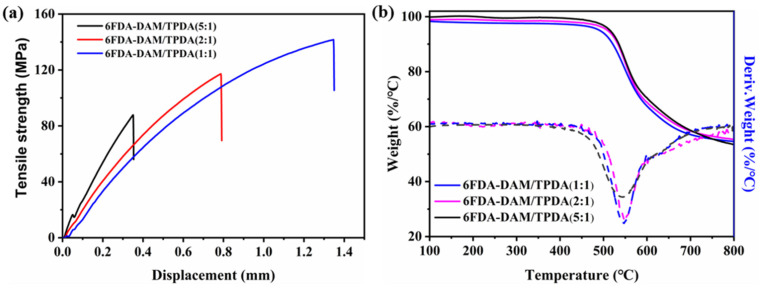
Mechanical properties (**a**) and TGA curves (**b**) of 6FDA−DAM/TPDA membranes.

The mechanical properties of 6FDA-DAM/TPD membranes are presented in Table 1 and Figure 3a. The membranes cast using the low-temperature method exhibited excellent mechanical properties because of the formation of robust three-dimensional networks. Among the membranes, the 6FDA−DAM/TPDA (5:1) membrane with the lowest DAM content showed relatively lower mechanical strength. However, it still exhibited a tensile strength of 87.8 MPa and an elongation at a break of 3.5%, which are sufficiently high for gas permeation measurements and among the highest reported values for high-performance membranes [27,28,29]. The thermal stability of the 6FDA−DAM/TPDA polyimides was determined by thermal gravimetric analysis (TGA), as shown in Table 1 and Figure 3b. As expected, all three 6FDA−DAM/TPDA networks exhibited excellent thermal stability. The decomposition temperature at 5% weight loss (T_d,5%_) ranged from 515 to 525 °C, and the temperature for 10% weight loss was even higher than 536 °C. Furthermore, all samples exhibited a char yield of at least 56% even after reaching 800 °C.

**Figure 4:**
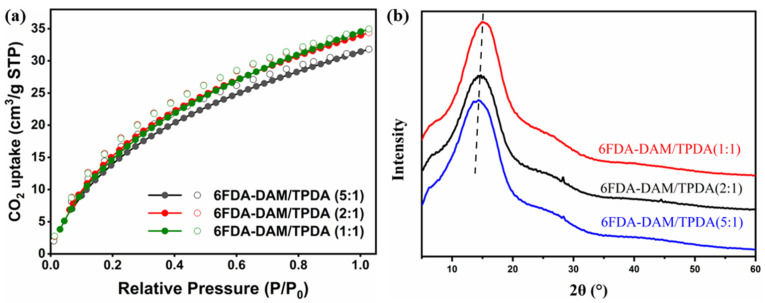
(**a**) CO_2_ adsorption–desorption isotherms of 6FDA−DAM/TPDA membranes at 273 K. (**b**) XRD patterns of 6FDA−DAM/TPDA membranes, and the samples with DAM/TPDA monomer ratios of 1:1, 2:1 and 5:1 are represented by red, black and blue lines, respectively.

To assess the porosity characteristics of the 6FDA−DAM/TPDA polymers, their BET specific surface areas were determined using CO_2_ as the probe gas at 273 K, as shown in Figure 4a. The 6FDA−DAM/TPDA with a DAM/TPDA ratio of 1:1 exhibited a BET surface area of 205 m^2^/g. When the DAM concentration was increased, the BET surface area increased to 223 m^2^/g for the 2:1 ratio and 227 m^2^/g for the 5:1 ratio. These results indicated that the porosity of the 6FDA−DAM/TPDA polymers was enhanced with an increasing DAM concentration.

The fractional free volumes (FFV) within the polyimide networks were calculated to evaluate the change in free volume (Table 2). Among the 6FDA−DAM/TPDA membranes, the 6FDA−DAM/TPDA (5:1) exhibited the highest FFV of 22.8%. As the DAM content decreased to 2:1 and 1:1, the FFV values decreased to 20.6% and 20.1%, respectively. This indicated that a higher concentration of DAM moiety led to a greater generation of FFV within the polyimides, as it extends the length of the linear unit around the crosslinking center. Furthermore, the tetramine TPDA-based polyimide networks presented in this study demonstrated higher FFV compared to previously reported HBPI membranes based on triamines, which possessed FFV values ranging from 17.3% to 18.7% [16]. This higher FFV in the 6FDA−DAM/TPDA membranes allows for increased diffusion of gas molecules into and out of the polymer networks, thereby facilitating gas transportation through the membranes.

Figure 4b and Table 2 illustrate the results of wide-angle X-ray diffractometry (WXRD) conducted to examine the chain packing efficiency of the 6FDA−DAM/TPDA membranes. The 2θ values for 6FDA−DAM/TPDA (1:1), 6FDA−DAM/TPDA (2:1), and 6FDA−DAM/TPDA (5:1) were measured at 14.9°, 14.7° and 14.4°, respectively. These values were then used to calculate the *d*-spacing, which represents the distance between polymer chains, using Bragg’s law. It is evident that the *d*-spacing value increased with an increase in DAM content. The 6FDA−DAM/TPDA (5:1) membrane exhibited the largest *d*-spacing of 6.16 Å, which was significantly larger than that of 6FDA−DAM/TPDA (1:1) with a *d*-spacing of 5.93 Å. This indicated that the presence of DAM comonomer reduced the crosslinking density and hindered the chain packing efficiency because of the steric hindrance caused by the three methyl groups. Moreover, the *d*-spacing of the tetramine-based 6FDA−DAM/TPDA membranes reported in this study was larger than that of the previously reported triamine-based HBPI networks [16]. This observation supports the notion that these tetramine-based HBPI have more loosely packed polymer chains compared to the reported triamine-based HBPI networks owing to the introduction of the tetramine crosslinking center and the DAM comonomer.

### 3.3. Gas Permeation Properties

Table 3 provides the pure gas permeation properties of the 6FDA−DAM/TPDA membranes. The results indicate that the gas permeabilities of the membranes increased as the DAM content increased, while the selectivities experienced only a minimal decrease. Specifically, when comparing 6FDA−DAM/TPDA (1:1) and 6FDA−DAM/TPDA (5:1), the CO_2_ permeability approximately doubled from 96.2 Barrer to 190.2 Barrer, whereas the ideal selectivity of CO_2_ over N_2_ lightly decreased from 21.9 to 19.5. The O_2_ permeability of 6FDA−DAM/TPDA (5:1) (40.8 Barrer) was more than twice as high as that of 6FDA−DAM/TPDA (1:1) (19.0 Barrer), with a negligible change in the corresponding O_2_/N_2_ selectivity values. The improvement of gas permeability suggests that a higher concentration of DAM leads to a longer length of the linear crosslinker, resulting in bigger microporous sizes that favor gas permeabilities. Typically, higher gas permeability is accompanied by poorer gas pair selectivity, as they are considered to have a trade-off relationship [9]. In this study, the gas permeabilities of the 6FDA−DAM/TPDA membranes improved with an increase in the DAM/TPDA ratio, while the gas selectivities remained nearly unchanged. Freeman proposed that the higher polymer chain rigidity contributes to better gas selectivity [9].The observed consistent gas selectivity in current study can be ascribed to the high rigidity nature of the hyperbranched network structure.

For comparative purposes, Table 3 also includes gas permeability data from previously reported triamine-based polyimide membranes and some commercial membranes [16]. When compared, the 6FDA−DAM/TPDA membranes constructed with the tetramine crosslinking center exhibited significantly higher CO_2_ permeability (ranging from 96.2 to 190.2 Barrer) compared to the triamine-based membranes, such as MN-PI-XS24 (37.4 Barrer) [21], 6FDA-TAPB (47 Barrer) [19], and 6FDA-TAPA-TPA (65 Barrer) [14]. For example, 6FDA−DAM/TPDA (5:1) demonstrated a CO_2_ permeability of 190.2 Barrer, which was five times higher than that of MN-PI-XS24 (37.4 Barrer). As expected, the CO_2_/N_2_ selectivity of 6FDA−DAM/TPDA was lower compared to MN-PI-XS24 owing to the trade-off relationship between gas permeability and selectivity. A similar trend was observed in O_2_/N_2_ gas pair separation. We suppose that the tetramine-based crosslinking network, combined with the addition of the rigid DAM comonomer, generated larger micropores which facilitated the transport of gas molecules through membranes. As a result, tetramine-based HBPI membranes generally exhibited higher permeability than triamine-based membranes. This observation is further evidenced by the increased *d*-spacing and FFV values for 6FDA−DAM/TPDA (5:1) compared to MN-PI-XS24.

To explore the impact of changing the crosslinking center from triamine to tetramine and incorporating the rigid DAM comonomer on the gas separation performance of 6FDA−DAM/TPDA membranes, the diffusion coefficient and solubility coefficient were analyzed and are summarized in Table 4 and Table 5. It is evident that increasing DAM loading in 6FDA−DAM/TPDA membranes resulted in higher diffusion coefficients while the solubility coefficients showed no significant increase. This confirms that the existence of rigid DAM was more microporous which favored gas permeabilities. Furthermore, when comparing with the previously reported triamine-based MN-PI-XS24, both the diffusion coefficient and solubility coefficient of the tetramine-based 6FDA−DAM/TPDA membranes were simultaneously enhanced [16]. This suggests that the high permeability of the 6FDA−DAM/TPDA membrane is not only attributed to the high gas diffusivity (*D*) governed by a high inter-chain separation but also the improved gas solubility (*S*) resulting from increased acid-base interaction between CO_2_ gas molecules and nitrogen atoms of polymer skeleton.

**Figure 5:**
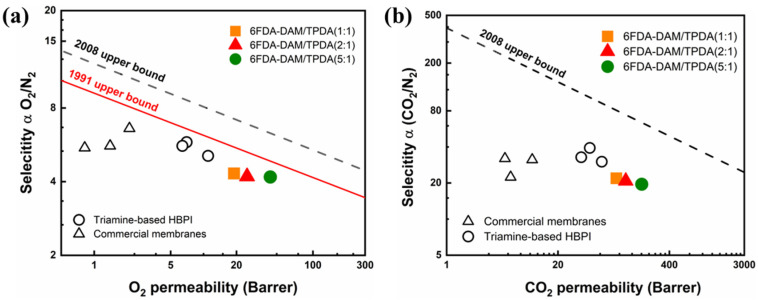
Robeson plots for (**a**) O_2_/N_2_ (**b**) CO_2_/N_2_ gas pairs showing the gas permeability data for 6FDA−DAM/TPDA membranes with monomer ratio of 1:1 (■), 2:1 (▲) and 5:1 (●). For the purposes of comparison, some previously reported data are shown for triamine-based HBPI membranes (○) and commercially used membranes (∆).

When compared to commercially used membranes such as Cellulose, Matrimid^®^ and Polysulfone in Robeson upper bounds [5,30,31], 6FDA−DAM/TPDA membranes displayed outstanding O_2_ permeation along with good O_2_/N_2_ selectivities, positioning their overall gas separation performance much closer to the 1991 Robeson upper bounds for O_2_/N_2_ (Figure 5a). The data points for tetramine-based 6FDA−DAM/TPDA membranes and triamine-based membranes appeared at a similar distance from the upper bound, but 6FDA−DAM/TPDA membranes were more permeable than the triamine-based membranes. This advantage allowed the 6FDA−DAM/TPDA membranes to minimize the required footprint of the membrane system in practical applications. A similar behavior was observed for the CO_2_/N_2_ gas pair, compared with the commercial membranes, where 6FDA−DAM/TPDA membranes exhibited higher CO_2_ permeabilities and comparable CO_2_/N_2_ selectivities, placing them closer to the 2008 CO_2_/N_2_ upper bound (Figure 5b). In comparison to MN-PI-XS24, 6FDA−DAM/TPDA membranes had a similar distance to the upper bound but with higher permeability and lower selectivity.

### 3.4. Anti-Aging Properties

**Figure 6:**
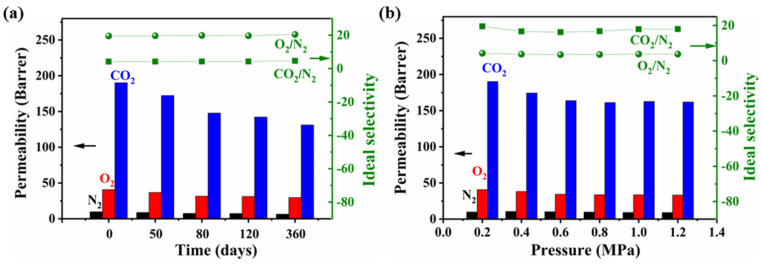
(**a**) Gas permeabilities and selectivities of 6FDA−DAM/TPDA (5:1) membrane after aging for different days. (**b**) Pressure-dependent gas permeabilities and selectivities for 6FDA−DAM/TPDA (5:1) membrane.

The physical aging of glassy polymer membranes poses a significant challenge to their practical application as it leads to a decrease in permeability over time owing to the loss of free volume [8]. To assess the anti-aging properties of 6FDA−DAM/TPDA membranes, the change in gas permeability was monitored over a period of 360 days (Figure 6a and Table S2). Considering the overall gas separation performance discussed earlier, the 6FDA−DAM/TPDA membrane with a 5:1 ratio was specifically selected for the investigation of physical aging and anti-plasticization. After an initial 80-day period, the CO_2_ permeability of 6FDA−DAM/TPDA (5:1) decreased from 190.2 to 147.8 Barrer, experiencing a 22.3% reduction. Subsequently, the permeability remained stable until the end of the 360-day period. Overall, the membrane retained 69% of its initial CO_2_ permeability, accompanied by a slight increase in CO_2_/N_2_ selectivity. In terms of O_2_ permeability, the 6FDA−DAM/TPDA (5:1) membrane exhibited superior anti-aging properties, retaining 73% of its initial O_2_ permeability after 360 days of aging.

In contrast, representative linear polyimide membranes suffered from more pronounced physical aging issues. For instance, PIM-PI-EA retained only 44% of its CO_2_ permeability and 48% of its O_2_ permeability after 372 days of aging [32]. Another example, SBFDA-DMN, maintained just 10% of its CO_2_ permeability and 13% of its O_2_ permeability after a shorter period of 200 days [33]. These findings highlight the superior aging resistance of 6FDA−DAM/TPDA (5:1) membranes. This advantage can be ascribed to their hyperbranched network structure, which efficiently restricts the relaxation of the polymer chain.

### 3.5. Pressure-Dependent Gas Separation Performance

Another major challenge in the practical application of membrane-based gas separation is plasticization, which refers to the swelling of the membrane when it absorbs condensable gases, such as CO_2_, at elevated pressures [34,35]. This phenomenon leads to lower selectivity and increased permeability. To assess the plasticization resistance of 6FDA−DAM/TPDA (5:1) membranes, the gas permeability was measured at feed pressure ranging from 0.2 to 1.2 MPa (Figure 5b and Table S3). The results indicated that the CO_2_ permeability of 6FDA−DAM/TPDA (5:1) membrane exhibited a slight decrease as the feed pressure increased from 0.2 MPa to 0.6 MPa. Once the pressure reached 0.6 MPa, the permeability remained stable even as the pressure further increased to 1.2 MPa. The ideal gas selectivity for CO_2_/N_2_ also experienced a slight reduction from 19.51 to 17.97 as the upstream pressure increased from 0.2 MPa to 0.6 MPa. Based on these findings, it can be concluded that no significant plasticization phenomenon was observed in 6FDA−DAM/TPDA (5:1) until the feed pressure reached 1.2 MPa. This suggests that the membrane exhibits good resistance to plasticization under typical operating conditions.

## 4. Conclusions

To summarize, this study describes the preparation and characterization of novel tetramine-based hyperbranched polyimide membranes with varying DAM/TPDA monomer ratios. The resultant membranes displayed remarkable thermal stability, and the formation of a crosslinked network structure was successfully verified. The inclusion of the tetramine crosslinking center and DAM comonomer resulted in enhanced fractional free volume (FFV) and improved porosity in the membranes. Gas permeation measurements revealed that the 6FDA−DAM/TPDA membranes outperformed both the triamine-based HBPI membrane and commercially used membranes in terms of gas permeability, while maintaining comparable gas pair selectivity. Additionally, these membranes exhibited excellent resistance to physical aging and plasticization under high feed pressure conditions, which can be attributed to their advanced three-dimensional crosslinking structure. Overall, this study presents a novel approach to enhance gas permeability in hyperbranched polyimide membranes without compromising gas pair selectivity and physical stability.

## Figures and Tables

**Table 1:** Mechanical and thermal properties of 6FDA−DAM/TPDA membranes.

Membrane	Tensile Strength (MPa)	Elongation at Break (%)	T_d,5%_ (°C)	T_d,10%_ (°C)	Char Yield (%)
6FDA−DAM/TPDA(1:1)	141.6	13.5	525	541	56
6FDA−DAM/TPDA(2:1)	117.2	7.9	515	536	57
6FDA−DAM/TPDA(5:1)	87.8	3.5	517	538	57

**Table 2:** Density, 2θ, FFV and BET values of 6FDA−DAM/TPDA membranes.

Membrane	Density (g/cm^3^)	FFV(%)	2θ(◦)	*d* Spacing (Ǻ)	BET (m^2^/g)
6FDA−DAM/TPDA(1:1)	1.319	20.1	14.9	5.93	205
6FDA−DAM/TPDA(2:1)	1.311	20.6	14.7	6.02	223
6FDA−DAM/TPDA(5:1)	1.274	22.8	14.4	6.16	227

**Table 3:** Pure gas permeability and selectivity of 6FDA−DAM/TPDA membranes measured at 30 °C and a feed pressure of 0.2 MPa.

Membrane	Permeability (Barrer) ^a^			Ideal Selectivity (*α*)		Ref.
	N_2_	O_2_	CO_2_	O_2_/N_2_	CO_2_/N_2_	
6FDA−DAM/TPDA(1:1)	4.4	19.0	96.2	4.3	21.9	This work
6FDA−DAM/TPDA(2:1)	5.9	25.0	123.6	4.2	20.8	This work
6FDA−DAM/TPDA(5:1)	9.8	40.8	190.2	4.2	19.5	This work
MN-PI-XS24	1.14	6.41	37.4	5.6	32.8	[21]
6FDA-TAPB	1.2	7.0	47.0	5.8	39.2	[19]
6FDA-TAPA-TPA	2.16	11	65.0	5.1	30.1	[14]
Cellulose	0.15	0.82	4.8	5.5	32.0	[30]
Matrimid^®^	0.32	2.1	10	6.6	31.3	[5]
Polysulfone	0.25	1.4	5.6	5.6	22.4	[31]

^a^ 1 Barrer = 10^−10^ [cm^3^ (STP) cm]/(cm^2^ s cmHg).

**Table 4:** The pure gas diffusion coefficients and the ideal diffusion selectivity of membranes.

Membrane	D (10^−8^ cm^2^/s)			Ideal Selectivity (*α*)		Ref.
	N_2_	O_2_	CO_2_	O_2_/N_2_	CO_2_/N_2_	
6FDA−DAM/TPDA(1:1)	3.13	4.18	8.82	1.33	2.62	This work
6FDA−DAM/TPDA(2:1)	4.04	5.41	11.1	1.34	2.76	This work
6FDA−DAM/TPDA(5:1)	5.77	7.94	16.0	1.37	2.78	This work
MN-PI-XS24	1.10	4.64	4.29	4.22	3.90	[21]

**Table 5:** The pure gas solubility coefficients and the ideal solubility selectivity of membranes.

Membrane	S (10^−2^ cm^3^/(cm^3^cmHg))			Ideal Selectivity (*α*)		Ref.
	N_2_	O_2_	CO_2_	O_2_/N_2_	CO_2_/N_2_	
6FDA−DAM/TPDA(1:1)	1.41	4.54	10.9	3.22	7.73	This work
6FDA−DAM/TPDA(2:1)	1.47	4.62	11.1	3.14	7.55	This work
6FDA−DAM/TPDA(5:1)	1.69	5.14	11.9	3.04	7.04	This work
MN-PI-XS24	1.03	1.38	8.71	1.34	8.46	[21]

## Data Availability

Data are contained within the article.

## Supplementary Materials

The following supporting information can be downloaded at: https://www.mdpi.com/article/10.3390/polym15143017/s1. Electronic supplementary information (ESI) available: Table S1: Gel content of 6FDA−DAM/TPDA membranes in different solvents; Figure S1: Physical appearances of 6FDA−DAM/TPDA membranes with varied DAM contents; Table S2. Gas permeabilities and selectivities of the 6FDA−DAM/TPDA (5:1) membrane after aging for different days; Table S3. Pressure-dependent gas permeabilities and selectivities for the 6FDA−DAM/TPDA (5:1) membrane; The calculation method of gas diffusion and solubility coefficients.
