# Supplementary material for: Tetramine-Based Hyperbranched Polyimide Membranes with Rigid Crosslinker for Improved Gas Permeability and Stability
Source: Polymers (Basel). 2023 Jul 12;15(14):3017. doi: 10.3390/polym15143017 (PMC10386325; doi:10.3390/polym15143017)
Supplement: Supplementary file 1 [file polymers-15-03017-s001.zip › polymers-2489750-supplementary.pdf]

Supporting Information

Tetramine-based Hyperbranched Polyimide Membranes with Rigid Crosslinker for Improved Gas Permeability and Stability

Xiangyun Liu [1,2], Honglei Ling [2,*], Jiangzhou Luo [2], Xueping Zong [2] and Song Xue [2,*]

[1] School of Materials Science and Engineering, Tianjin University of Technology, Tianjin 300384, China
[2] Tianjin Key Laboratory of Organic Solar Cells and Photochemical Conversion, School of Chemistry and Chemical Engineering, Tianjin University of Technology, Tianjin 300384, China
* Correspondence: linghl@email.tjut.edu.cn (H.L.); xuesong@ustc.edu.cn (S.X.)

Table S1. Gel content of 6FDA-DAM/TPDA membranes in different solvents.

Sample	Gel content (%)		
	THF	DCM	DMF
6FDA-DAM/TPDA(1:1)	99.92	99.89	99.90
6FDA-DAM/TPDA(2:1)	99.97	99.96	99.93
6FDA-DAM/TPDA(5:1)	99.98	99.96	99.93

Note: The acronyms for THF, DCM and DMF represent tetrahydrofuran, dichloromethane, and N,N-dimethylformamide, respectively.

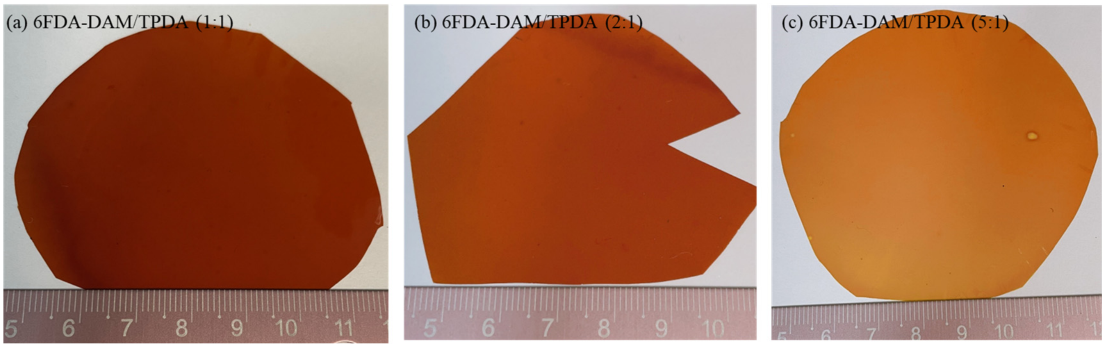

Figure S1. Physical appearances of 6FDA-DAM/TPDA membranes with varied DAM contents.

Table S2. Gas permeabilities and selectivities of the 6FDA-DAM/TPDA(5:1) membrane after aging for different days.

Time (days)	P (Barrer)			Ideal selectivity (α)	
	N_2	O_2	CO_2	O_2/N_2	CO_2/N_2
0	9.75	40.78	190.2	4.18	19.51
50	8.76	36.7	172.3	4.19	19.67
80	7.46	31.63	147.8	4.24	19.82
120	7.33	31.22	142.2	4.26	19.71
360	6.43	29.77	131.2	4.63	20.41

Table S3. Pressure-dependent gas permeabilities and selectivities for the 6FDA-DAM/TPDA(5:1) membrane.

Pressure (MPa)	P (Barrer)			Ideal selectivity (α)	
	N_2	O_2	CO_2	O_2/N_2	CO_2/N_2
0.2	9.75	40.78	190.2	4.18	19.51
0.4	10.48	38.15	174.3	3.64	16.63
0.6	10.12	34.35	163.8	3.39	16.19
0.8	9.63	33.68	161.2	3.49	16.74
1.0	9.11	33.66	162.7	3.69	17.86
1.2	9.01	33.19	161.9	3.68	17.97

The gas diffusion and solubility coefficients calculation:

The pure gas diffusion coefficient (10^{-8}cm^2/s) was calculated by the equation of $D = l^2/6\theta$, where θ is the time-lag of the permeability measurement. Ideal diffusion selectivity(α) for a gas pair (A/B) is calculated as the ratio of their pure-gas diffusion coefficients:

$$\alpha_{A/B} = \frac{D_A}{D_B}$$

The solubility coefficient S (10^{-2}cm^3/(cm^{3}cmHg)) was obtained via the diffusion-solution model using the formula of $S = P/D$. Ideal solubility selectivity(α) for a gas pair (A/B) is calculated as the ratio of their pure-gas solubility coefficients:

$$\alpha_{A/B} = \frac{S_A}{S_B}$$
